# Book Review: Recreios Collegiaes

**DOI:** 10.3389/fpsyg.2020.587942

**Published:** 2020-09-18

**Authors:** Mário Duarte Maia Rodrigues

**Affiliations:** Agrupamento de Escolas da Lousã, Lousã, Portugal

**Keywords:** traditional games, Pedro Aloy, motor praxiology, education, physical education (P.E.)

This book is one of the oldest and most important publications on the topic of traditional games in Portugal. The author, Pedro Aloy, was a Jesuit priest born on the 23rd of May 1840 in Mallorca, Spain. Before and after his ordainment as a priest, Aloy taught at the Colégio de Campolide, an all-boys Jesuit college in Portugal, and served as its subdirector for 12 years.

The book was written for his students as a guide for their leisure time and follows the pedagogical ideology of Jesuit priests, pioneers of education in Portugal, of introducing physical activities and games in schools (Ferreira and Ferreira, 2003). Thematically, the book focuses on traditional games and is an example of how Jesuits, always ahead of their peers on pedagogy, highlighted the benefits of physical exercise toward our physical and mental health and well-being. Because it was written in the late nineteenth century, when education was mostly a male privilege, the book contains dated concepts and language. Nevertheless, most games included in the book work equally well in modern schools with mixed-gender student populations.

In the opening chapter, titled “Advertências” (Introduction), the author explains and highlights the importance of his work: “We intended to simply collect a series of pleasant activities for our students, so that they could enjoy a more moral, healthier, and happier collegial life during leisure hours.” We note the author's particular care in recognizing that “the playground is a simulacrum of the students' freedom,” that is, that the playground is like a laboratory, a place where students learn social and physical skills through experimentation. The choice of which traditional games to include in the book, as well as their distribution throughout the school year, sought to keep the students happy, motivated, and avoided the “fervor of the game,” which could lead to disorder. In this way, the author tried to find a balance between playing games and maintaining the students' spontaneity and individual freedom.

As a teacher, Aloy proposes a very practical organization of the games according to the periods of the school year— “winter,” “spring,” and “summer.” He then further subdivides them in two categories, depending on the spatial needs of the playground: games with more action and movement (“jogos principaes”), that required larger spaces, and games with little to no action, that could be played anywhere (“jogos accessórios”).

Throughout the book, the author describes a total of 226 games, including variants, which is far more than the 150 listed originally. Of these 226, 170 are motor games, 32 are social games, and 23 are tabletop games. The 170 motor games, which we consider more relevant for modern physical education, can further be subdivided in competitive (79) or non-competitive (91) games. This diversity of games stands in contrast with current practices in physical education, that tends to focus only on competitive games and sports such as football, basketball, or athletics.

We applied the concepts of motor praxeology to analyze and understand the educational principles behind the 170 motor games included in this work (Parlebas, 2001; Lagardera and Lavega, 2003). According to this scientific discipline, each game has an internal logic, or identity card, that challenges players to solve four types of internal relationships: with others, with the physical space, with time, and with the materials.

For the relationship with others, we observe a clear preference for team or socio-motor games (*n* = 134; 78.8%) over individual or psycho-motor games (*n* = 36; 21.2%). The book promotes a diverse and relational physical education through a multitude of games that encourage different types of interpersonal relationships: cooperation (e.g., dancing) and opposition (e.g., duels, chases), as well as cooperation-opposition (e.g., symmetrical and asymmetrical team duels and ambivalent games, including paradoxical games).

Regarding the relationship with the physical space, we find a majority of games played in stable spaces (*n* = 123; 72.4%), during which the conditions remain constant. The remaining games (*n* = 47; 27.6%) are played in unstable spaces, leading to unforeseen consequences. This preference for games played in stable spaces is understandable given that schools have to ensure optimal and controllable playing conditions: stables spaces provide easier means of control.

As for the relationship with time, we observe that there is a slight majority of games without competition (*n* = 91; 53.5%), in which playing the game is the end goal, vs. those with competitive elements (*n* = 79; 46.5%), in which there are winners and losers. This balance fosters a more inclusive and less discriminating environment for all participants.

Finally, for the relationship with the materials, we find that the majority of games utilize some sort of physical material (*n* = 133; 78.2%), compared to those that do not (*n* = 37; 21.8%). Further, we are able to deduce that the materials used in these games are quite diverse and often originate from the students' surroundings—either naturally or purposely-built—therefore promoting environmentally sustainable educational practices.

In summary, this is an unique body of work in Portugal, presenting itself as an educational project that highlights traditional games, and referring the reader to a time where physical education was introduced as a pedagogical tool. This book encompasses a holistic perspective of our education and demonstrates that physical and relational acts are key to our socialization. Furthermore, despite having been written more than 100 years ago, this work shows particular care for inclusion, emotion, and democracy, all toward promoting the students' social well-being and even environmental sustainability. We believe the rich and balanced offer of games presented in this book is a valuable complement to the modern physical education of all genders.

## Author Contributions

MDMR wrote, reviewed, and supervised this work.

## Conflict of Interest

The author declares that the research was conducted in the absence of any commercial or financial relationships that could be construed as a potential conflict of interest.

## References

[B1] Ferreira J. V. Ferreira A.G. (2003). As práticas físicas, em Portugal, no Antigo Regime. Rev. Portuguesa Educ. Física 3, 56–63. Available online at: https://rpcd.fade.up.pt/_arquivo/artigos_soltos/vol.3_nr.3/Ferreira.pdf

[B2] Lagardera F. Lavega P. (2003). Introducción a la Praxiologia Motriz. Barcelona: Paidotribo.

[B3] Parlebas P. (2001). Juegos, Deporte y Sociedad. Léxico de Praxiologia Motriz. Barcelona: Paidotribo.

